# Systemic localization of seven major types of carbohydrates on cell membranes by dSTORM imaging

**DOI:** 10.1038/srep30247

**Published:** 2016-07-25

**Authors:** Junling Chen, Jing Gao, Min Zhang, Mingjun Cai, Haijiao Xu, Junguang Jiang, Zhiyuan Tian, Hongda Wang

**Affiliations:** 1 State Key Laboratory of Electroanalytical Chemistry, Changchun Institute of Applied Chemistry, Chinese Academy of Sciences, Changchun, Jilin 130022, P.R. China; 2 University of Chinese Academy of Sciences, Beijing 100049, P.R. China; 3 School of Chemistry and Chemical Engineering, University of Chinese Academy of Sciences, Beijing 100049, China

## Abstract

Carbohydrates on the cell surface control intercellular interactions and play a vital role in various physiological processes. However, their systemic distribution patterns are poorly understood. Through the direct stochastic optical reconstruction microscopy (dSTORM) strategy, we systematically revealed that several types of representative carbohydrates are found in clustered states. Interestingly, the results from dual-color dSTORM imaging indicate that these carbohydrate clusters are prone to connect with one another and eventually form conjoined platforms where different functional glycoproteins aggregate (*e.g.*, epidermal growth factor receptor, (EGFR) and band 3 protein). A thorough understanding of the ensemble distribution of carbohydrates on the cell surface paves the way for elucidating the structure-function relationship of cell membranes and the critical roles of carbohydrates in various physiological and pathological cell processes.

Cells rely on their ability to interact with neighboring cells and the extracellular environment. The surfaces of most types of cells are covered by a carbohydrate layer consisting of membrane glycoproteins and glycolipids. Owing to the localization and structural diversity, cell surface carbohydrates intrinsically encode cell-cell recognition factors for directing intercellular interactions during embryonic development^1^, the binding of pathogens to their target tissues^2^,^3^ and interactions among cells in the immune system^4^,^5^. In addition, alterations in the expression of cell-surface carbohydrates may lead to defects in cell-cell recognition or to uncontrolled cell growth and motility, which is related to neoplastic transformation and metastasis^6^,^7^. Thus, a better understanding of the molecular basis of cell-surface carbohydrates may reveal significant interventions in many areas of biology and medicine^7^,^8^.

Meanwhile, owing to ever-increasing awareness of the complexity of cell membranes and efforts to explore the structure and function of the plasma membrane, various models of the plasma membrane have been developed^9^,^10^,^11^,^12^. Accumulating evidence has indicated that the plasma membrane, with densely and heterogeneously distributed proteins, is laterally compartmentalized, suggesting the distribution of various membrane microdomains with nanoscale organization on the plasma membrane. Although most cell-surface proteins are virtually glycosylated, the role of carbohydrates in the organization of the plasma membrane is not considered in these models. In fact, carbohydrate-based interactions play a crucial role in the organization of cell membranes. For example, the spatial pattern of carbohydrates controls the phase behavior of multiphase model lipid membranes^13^, and glycan-based connectivity contributes to the stability of cell membranes^14^. Thus, a thorough understanding of the ensemble distribution of carbohydrates on the cell surface is highly desired to illuminate the structure-function relationship of cell membranes. Although considerable efforts have been made toward the imaging and tracking of carbohydrates in recent years^15^,^16^,^17^,^18^,^19^, details regarding the morphological features of various carbohydrates on the cell membrane and the relationship between the distribution of carbohydrates and the organization of cell membranes have remained largely unexplored.

Recently, single-molecule-localization-based super-resolution imaging techniques have demonstrated unparalleled advantages in visualizing subcellular features with previously unprecedented detail, providing a powerful strategy for mapping the nanoscale organizations of membrane molecules^12^,^20^,^21^. In our previous study using dSTORM to measure carbohydrate patterns on cell membranes, we have demonstrated that N-acetylglucosamine (GlcNAc) exists in irregular clusters on the apical membrane, and most of these clusters colocalize with lipid rafts^22^. Here, we focused on the following critical issues regarding the organization of carbohydrates: (1) whether other types of representative carbohydrates are involved in the formation of these clusters; (2) the spatial relationship between different types of carbohydrates at the cellular membrane; and (3) the localized relationship between carbohydrates and different membrane functional proteins. With these questions in mind, we systematically investigated the distribution of seven types of representative carbohydrates on the Vero apical membranes using the dSTORM technique and revealed the spatial distribution relationships between carbohydrates of interest and GlcNAc, and the relationships between the carbohydrates and membrane proteins (EGFR and band 3).

## Results

### dSTORM imaging and cluster analysis of seven types of representative carbohydrates on Vero apical membranes

To ensure that all the carbohydrates on the membrane could be labeled, the saturated labeling concentration of the Alexa647-linked lectin was determined by plotting the labeling curves before characterizing the distribution patterns of the carbohydrates on Vero membranes (Fig. S1, in Supporting Information). Based on the curves, the labeling concentration of each lectin was determined as ~3 μg/mL of maackia amurensis lectin (MAL) showing high affinity for sialic acid linked to galactose by an α2-3 linkage (Sia)^23^,^24^, ~9.3 μg/mL of lectin from Phaseolus vulgaris (PHA-L) for oligosaccharide (pentasaccharide sequence Galβ1-4GlcNAcβ1-2(Galβ1-4GlcNAcβ1-6) Manα1-R (the so-called “2,6-branch”))^25^,^26^, ~2.88 μg/mL of wheat germ agglutinin (WGA) that mainly interacts with high affinity with N-acetyl-D-glucosamine (GlcNAc) and its β-(1 → 4)-linked oligosaccharides^25^,^27^, ~12.5 μg/mL of lectin from Anguilla anguilla (eel) (AAA) that is a fucose (Fuc) specific lectin^25^,^28^, ~7 μg/mL of lectin from Morniga M (MNA-M) whose binding specificities of mannose (Man)^29^, ~3.75 μg/mL of erythrina cristagalli lectin (ECL) for D-Galβ1-4GlcNAc (Gal)^25^, and ~10.5 μg/mL of lectin from glycine max (SBA) possessing high affinity for N-acetylgalactosamine (GalNAc)^27^,^30^.

To avoid artificial clusters formed by cross-linking of lectins when labeling carbohydrates, we performed a series of imaging experiments of GlcNAc on live and fixed Vero apical membranes with increasing fixing time to compare the morphological changes of carbohydrates under the effect of the cross-linking of lectins. Novel views about the structure of cell membranes indicate that lipids or proteins cannot freely move in the membrane as we expected (for example, the protein layer–lipid–protein island (PLLPI) model of cell membranes pointed out that the proteins on the ectoplasmic side of the cell membrane form a dense protein layer on the top of a lipid bilayer^10^,^31^; Engelman D. M. also supported that the membrane has higher protein occupancy than generally considered^32^; and it is confirmed that a hydrophobic membrane–spanning part of the protein is stiff with no appreciable internal flexibility^33^); therefore, the molecules (lipids and proteins) movement in cell membranes is extremely restricted. Based on our results, carbohydrates on the sufficiently fixed Vero apical membrane with a suitable fixing time can effectively avoid the cross-linking of lectins (Fig. S2, in Supporting Information).

**Figure 1:**
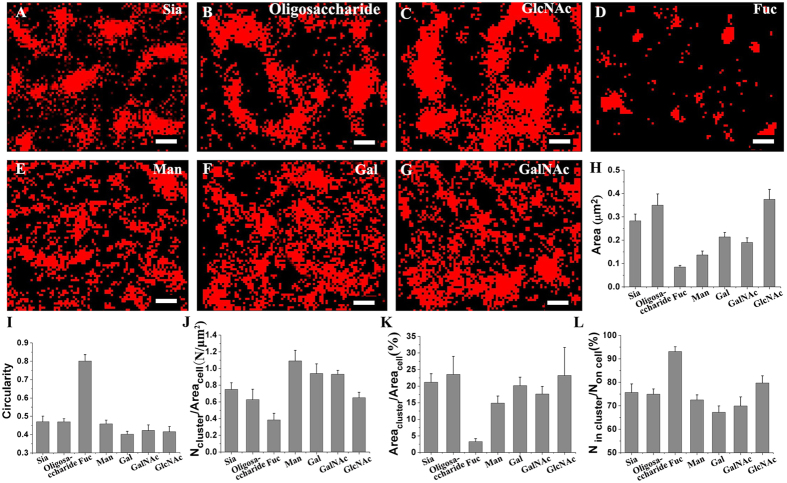
dSTORM imaging and cluster analysis of seven types of carbohydrates on Vero apical membranes labeled with their specific Alexa647-connected lectins. (**A–G**) dSTORM images of the organization of different carbohydrates (Sia (**A**), oligosaccharide (**B**), GlcNAc (**C**), Fuc (**D**), Man (**E**), Gal (**F**), and GalNAc (**G**)) on Vero apical membranes, depicting sharply distributed patterns of carbohydrates at nanoscale resolution. Scale bars are 500 nm. (**H,I**) Histograms of the average cluster area and the circularity of all types of carbohydrate clusters. (**J–L**) Histograms of the cluster density on unit cell membranes (**J**), coverage percentage of clusters on membranes (**K**), and the ratio of the number of localizations in clusters to the total localizations on the entire membrane (**L**), which together show the distribution features of the clusters on the entire membrane. All statistical analyses are acquired from more than ten cells (mostly 10–20 cells) in three independent experiments.

Using Alexa647-lectin at saturated labeling concentrations, we revealed the spatial organizations of seven types of representative carbohydrates on fixed Vero apical membranes at the nanometer level via dSTORM. Compared with conventional fluorescent imaging (upper left in Fig. S3A–G, Supporting Information), dSTORM imaging (the main parts in the upper images of Fig. S3A–G, in Supporting Information) unequivocally displays the clustering feature of all types of carbohydrates with markedly improved resolution. The detailed carbohydrate-dependent organization patterns are shown in Fig. 1A–G from the box regions in Fig. S3. Sia and oligosaccharide (Fig. 1A,B) are found to aggregate into large and dense clusters, similarly to GlcNAc (Fig. 1C). In sharp contrast, Fuc exist in relatively small and sparse clusters (Fig. 1D), whereas Man, Gal and GalNAc mostly organize into clusters without clear boundaries (Fig. 1F,G).

Due to the fact of dSTORM data — large variations in cluster size and shape for single type of carbohydrate, it is difficult to apply the pair-correlation function (PCF) and Ripley’s K functions, which work better when there is only one type of cluster^34^, to accurately analyze our data. Thus, image-based method and Density Based Spatial Clustering of Applications with Noise (DBSCAN)^35^,^36^ were selected for cluster analysis to acquire morphological information on carbohydrate clusters. Firstly, we implemented these two methods to analyze the morphology of Fucs that was mostly distributed in small clusters and relatively regular shape, and obtained similar cluster size and density with no significant difference (P values of the two-tailed unpaired t-test were larger than 0.05), whatever the threshold of cluster size was set larger than 0.01 μm^2^ or 0.04 μm^2^ (Fig. S4, in Supporting Information), which strongly demonstrates that both methods are suitable for analysis of Fuc clusters. However, for the analysis of GlcNAc clusters which are quite large and in irregular shape, no matter what the threshold of search radius (ε) for cluster identification was (from 35 nm to 60 nm), DBSCAN analysis cannot accurately recognize and identify GlcNAc clusters (Fig. S5, in Supporting Information), indicating this method is not good for most types of carbohydrates that have relative large clusters in irregular shapes. Therefore, we applied the image-based method to analyze all classes of carbohydrates, which makes better to compare the same parameter among all types of carbohydrates. Simply, during the image-based analysis, the qualified clusters (>0.04 μm^2^) were extracted from a binary image generated from original dSTORM image by removing the outliers (Fig. S6A–C, in Supporting Information, more details in Material and Methods), which more clearly displays the characteristics of the clusters, including size, shape and cluster densities (Fig. S6D–J, in Supporting Information). The average cluster area (Fig. 1H) and average circularity (Fig. 1I) of all carbohydrate clusters were analyzed to compare cluster size and roundness. The average sizes of different carbohydrate clusters (Fig. 1H) decrease in the following order: GlcNAc (0.37 ± 0.04 μm^2^) > oligosaccharide (0.35 ± 0.05 μm^2^) > Sia (0.28 ± 0.03 μm^2^) > Gal (0.21 ± 0.02 μm^2^) > GalNAc (0.19 ± 0.02 μm^2^) > Man (0.14 ± 0.02 μm^2^) > Fuc (0.08 ± 0.01 μm^2^). For the circularity, however, a different order is observed, *i.e.*, Fuc (0.80 ± 0.03) > Sia (0.47 ± 0.03) ≈ oligosaccharide (0.47 ± 0.02) > Man (0.45 ± 0.02) > GalNAc (0.42 ± 0.03) > GlcNAc (0.41 ± 0.03) > Gal (0.40 ± 0.02). Informatively, these data imply that both GlcNAc and oligosaccharide tend to form large clusters, whereas GlcNAc clusters show more complex boundaries. Sia also gather into relatively large clusters with similar shapes to oligosaccharide. In contrast, Fuc aggregates into the smallest clusters with the highest roundness. Gal, GalNAc and Man assemble into clusters with medium size and complex boundaries. To gain insight into the overall distribution of clusters on the entire membrane, the cluster density on unit cell membranes (Fig. 1J) was evaluated. The Sia, oligosaccharide and GlcNAc clusters are regularly distributed on the membrane with relatively low densities of 0.75 ± 0.08, 0.63 ± 0.12, and 0.65 ± 0.06 N/μm^2^, respectively. In contrast, the clusters with relatively small sizes display higher cluster densities on the membranes, namely 1.09 ± 0.13, 0.94 ± 0.11 and 0.93 ± 0.05 N/μm^2^ for Man, Gal and GalNAc, respectively. However, Fuc is an exception, which mostly distribute into the smallest clusters with the lowest cluster density (0.38 ± 0.08 N/μm^2^). For the quantitative measurement of the coverage percentage of clusters on the membrane, namely the ratio of the total area of clusters to the area of the cell membranes (Fig. 1K), a similar trend to the average cluster area is observed. Sia, oligosaccharide and GlcNAc display similar coverage percentages (21.16 ± 2.56%, 23.52 ± 5.46% and 23.19 ± 8.46%). In contrast, Fuc displays the lowest percentage, 3.28 ± 0.88%, owing to their smallest cluster size and lowest cluster density. For Gal, GalNAc, and Man, with medium cluster areas, the comparatively higher cluster density contributes to a relatively high area percentage (20.14 ± 2.56%, 17.62 ± 2.29%, and 14.86 ± 2.13%, respectively). Furthermore, the cluster capability is scaled by the ratio of the number of cluster localizations to the total localizations on the entire membrane (Fig. 1L). Remarkably, 93.10 ± 2.05% of Fuc localized into clusters, indicating a higher proportion of the molecules in clustering state.; Sia, oligosaccharide, GlcNAc and Man have comparable clustering abilities, with 75.65 ± 3.61%, 74.94 ± 2.24%, 79.70 ± 3.07% and 72.50 ± 2.11%, respectively, whereas Gal and GalNAc, with lower values (67.18 ± 2.71% and 69.90 ± 3.84%), are both relatively weaker at clustering.

Altogether, our results demonstrate that all types of carbohydrates investigated herein are prone to concentrating into clusters on the cell membrane. Type-dependent distribution features of these carbohydrates are clearly observed, which also further excludes the cross-linking of lectins. If clusters were cross-linked by lectins, the size of the carbohydrate cluster labeled by lectins would positively correlate with the number of lectin binding sites. Thus, GalNAc and Sia clusters would be of comparable size because their specific lectins (SBA and MAL) have the same number of binding domains. However, GalNAc clusters are much smaller than Sia clusters and are similar to those of Gal labeled by ECL containing less binding domains (Fig. 1). Besides, early studies have revealed that many membrane proteins gather into domains^37^,^38^, which is indirectly consistent with our findings that carbohydrates are prone to distribute into clusters, given that more than half of all membrane proteins are glycosylated. For the cell surface glycoproteins, there was a broad tendency toward organizing into functional domains for specific cellular responses^39^,^40^,^41^. Significantly, our results indicate that, by forming specific microdomains for certain functions, cell-surface carbohydrate-based interactions can impart an additional organization layer to the cell membrane, which is a significant complement to the existing cell membrane model that does not consider the vital role of carbohydrates.

### Colocalization between GlcNAc and other carbohydrates by dual-color dSTORM imaging

**Figure 2:**
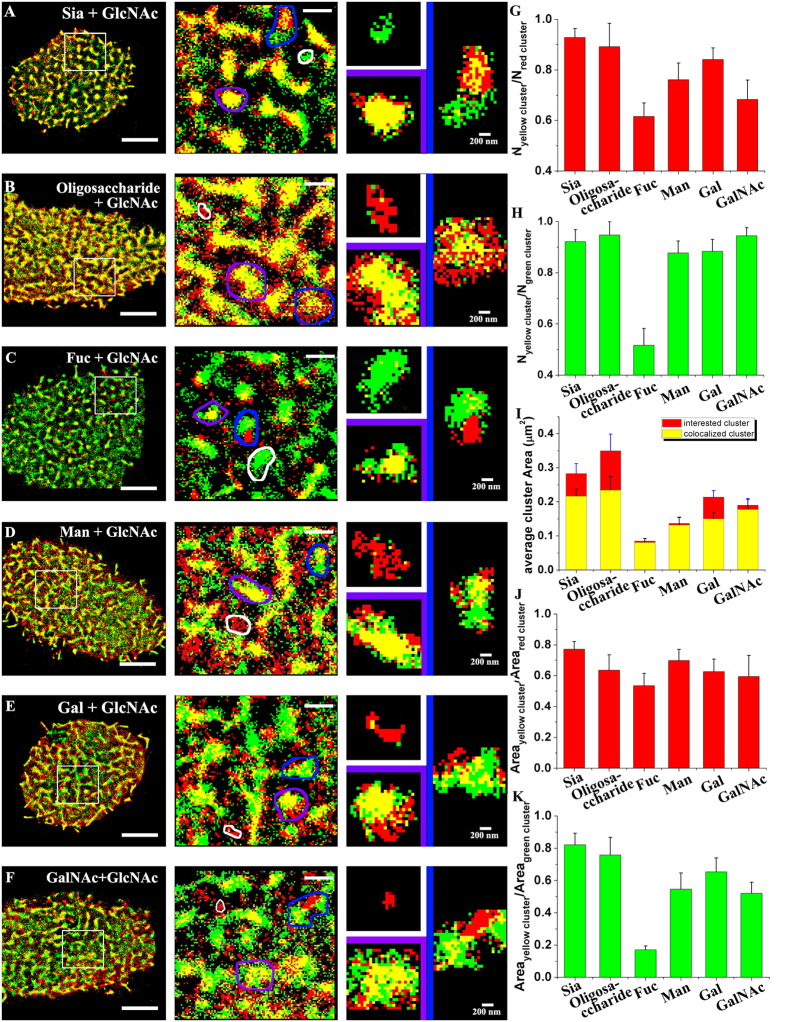
Dual-color dSTORM imaging of GlcNAc and other carbohydrates. (**A–F**, left) Representative superimposed images of the carbohydrates of interest (red color) and GlcNAc (green color) on Vero apical membranes, where the carbohydrates of interest are Sia (**A**), oligosaccharide (**B**), Fuc (**C**), Man (**D**), Gal (**E**) and GalNAc (**F**). The bars are 5 μm. (**A–F**, middle) The enlarged images of the boxed regions more clearly display the detailed spatial relationships between the carbohydrates of interest and GlcNAc. The bars are 1 μm. (**A–F**, right) Three classification types of clusters are shown: independent clusters (white box), colocalized clusters (purple box), and correlated clusters (blue box). The bars are 200 nm. (**G,H**) Histograms of the percentage of the number of colocalized clusters with respect to the total number of the carbohydrate clusters of interest (**G**) and of GlcNAc clusters (**H**). (**I**) Comparative histogram of the average cluster area of the colocalized regions (yellow columns) and the carbohydrate clusters of interest (red columns). (**J**,**K**) Distributions of the coverage percentage of the colocalized clusters with respect to the carbohydrate clusters of interest and the GlcNAc clusters. All statistical analyses are acquired from more than ten cells (mostly 10–15 cells) in three independent experiments.

Having characterized the distribution of the individual carbohydrates, we further investigated the spatial relationships of carbohydrate clusters. GlcNAc was selected as the reference in dual-color dSTORM imaging, owing to its large cluster size. By merging the dSTORM reconstruction images of GlcNAc (green in Fig. 2A–F) with the images of the carbohydrates of interest (red in Fig. 2A–F), we find that carbohydrate clusters of interest always colocalize with GlcNAc clusters, although relatively weak colocalization is observed in the case of Fuc due to its relatively small cluster size and low cluster density. According to the size of the colocalization zone, three classifications of spatial relationships are identified (Fig. 2A–F-right): correlated clusters (adjacent but with colocalization area <0.02 μm^2^, pink box), colocalized clusters (≥0.02 μm^2^, yellow box), and independent clusters (no association, white box). Closer observation of the enlarged images of the three classification groups of clusters reveals details of the distributed relationships of carbohydrate clusters with GlcNAc. To further characterize these relationships, systemic statistical analyses were performed. Based on the ratio of the total number of colocalization clusters to the total number of carbohydrate clusters of interest (Fig. 2G), 61.60 ± 0.05% of Fuc clusters, whose colocalization is less apparent in the superimposition, are found to colocalize with GlcNAc. Other carbohydrates display higher colocalization percentages: 92.80 ± 0.03% for Sia, 89.16 ± 0.09% for oligosaccharide, 76.13 ± 0.06% for Man, 84.14 ± 0.04% for Gal, and 68.30 ± 0.08% for GalNAc. Colocalization of the GlcNAc clusters (Fig. 2H) is similar: only 51.67 ± 0.06% of GlcNAc clusters colocalize with Fuc owing to the low ratio of the total number of Fuc clusters to the total number of GlcNAc clusters. In contrast, the percentages of GlcNAc clusters that colocalize with the other examined carbohydrates are high (92.12 ± 0.05% for Sia, 94.73 ± 0.05% for oligosaccharide, 87.72 ± 0.05% for Man, 88.39 ± 0.05% for Gal, and 94.42 ± 0.03% for GalNAc). Additionally, the overlapping degree of the two colocalized clusters is illustrated by a histogram of the average area of the colocalized portion (Fig. 2I). For Sia and oligosaccharide, the large colocalized areas (the yellow columns, 0.22 ± 0.02 μm^2^ and 0.23 ± 0.04 μm^2^) are smaller than the Sia and oligosaccharide clusters (the red columns) by 23.6% and 33.1%, indicating that these two carbohydrates are indeed overlaid by GlcNAc with a large colocalized region, but still leaving some regions uncorrelated to GlcNAc clusters with incomplete overlap. For Fuc and Man, no appreciable reduction in their colocalization areas is observed compared to their original size (decrease by 4.9% and 3.7%), suggesting that they locate inside GlcNAc clusters. In contrast to GalNAc (decrease by 7.3%), Gal displayed a relatively large decrease in colocalization area, 29.9%, owing to its larger original cluster size (0.21 ± 0.02 μm^2^) and a smaller colocalization area (0.15 ± 0.02 μm^2^). Furthermore, the percentage of total area of the colocalized clusters with respect to the total area of the carbohydrate clusters of interest (Fig. 2J) or the GlcNAc clusters (Fig. 2K) was statistically analyzed. The coverage percentages of colocalization regions for all types of clusters of interest are larger than 50%, suggesting that most of the carbohydrates of interest overlap with GlcNAc clusters. Similar results are obtained for the GlcNAc clusters, with the exception of Fuc. Specifically, only 17.1 ± 0.02% of GlcNAc clusters contribute to the colocalized regions due to the low area percentage in independent imaging, whereas more than 50% of the area of GlcNAc clusters colocalize with other types of carbohydrates. Thus, the colocalization features of these six carbohydrates with GlcNAc are verified by both the numerical percentage (Fig. 2G,H) and the area percentage (Fig. 2J,K) according to the statistical analysis.

Additionally, we also measured the Mander’s coefficient that is one intensity-based method to analyze the correlation between pixel intensities in two channels in conventional fluorescence imaging and super-resolution imaging^34^,^42^. From the result of Mander’s coefficient measurement (Fig. S7, Supporting Information), the examined carbohydrates and GlcNAc have high colocalization values (>0.6), expect the GlcNAc colocalized with Fuc (0.48). As a control, two randomized images representing two channels were created, and colocalization was tested by these two methods (Fig. S8, Supporting Information). Both the colocalized cluster percentage (29.59 ± 6.98% for channel 1 and 33.23 ± 9.6% for channel 2) and the Mander’s coefficient (0.11 ± 0.03 for channel 1 and 0.12 ± 0.04 for channel 2) were quite low, showing the high colocalization obtained from these two methods are real characteristics of our dSTORM data.

Based on the highly colocalized distributions of these carbohydrates on cell membranes, we infer that different types of carbohydrate clusters are not independent with each other, and they complementarily get together to form mesoscale functional platform where various glycoconjugates aggregate.

### Colocalization between the GlcNAc clusters and EGFR domains

**Figure 3:**
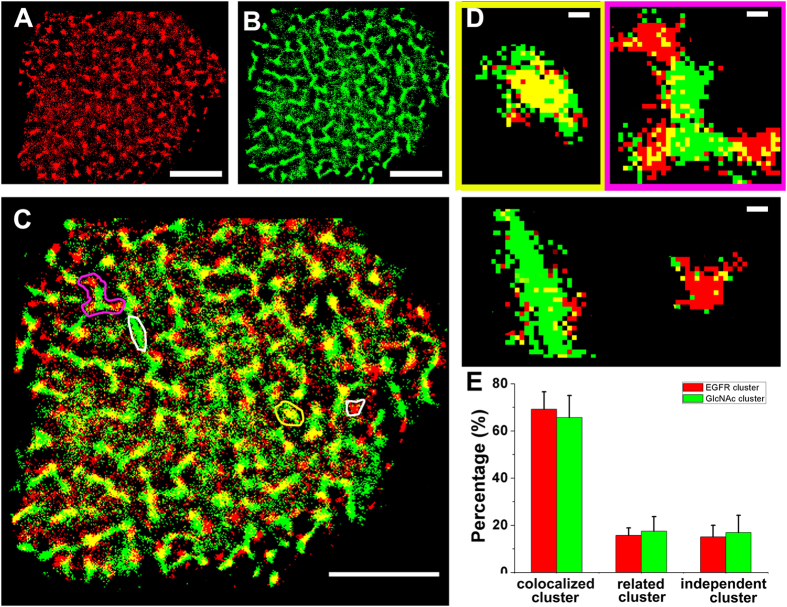
Dual-color dSTORM imaging of EGFR domains and GlcNAc clusters on Vero apical membranes. (**A,B**) Representative dSTORM reconstructed images of the nanoscale organization of EGFR (**A**) and GlcNAc (**B**) on Vero apical membranes, labeled with Alexa647-conjucated EGF and Alexa532-linked WGA, respectively. (**C**) In the superimposed images of (**A,B**), the colocalization (yellow region) is a clear feature. (**D**) The enlarged images represent three typical groups of clusters, the independent clusters (white box), the colocalized clusters (yellow box), and the correlated clusters (pink box). (**E**) Ratios of the cluster number of each group to the total number of EGFR clusters (red columns) and GlcNAc clusters (green columns). The bars are 5 μm in (**A–C**) and 200 nm in (**D**). The statistical analysis is acquired from ten cells in three independent experiments.

Considering the above-mentioned dual-color dSTORM imaging results, along with growing evidence of the nonhomogeneous distributions of most proteins that aggregate into various microdomains in the plasma membranes^38^,^41^,^43^, we raised the question of whether common carbohydrate clusters can provide a functional platform for the aggregation of proteins that mediates multiple biological functions on cell membranes. To address this question, EGFR was used as the model protein for its clustering distribution feature^44^ and glycosylation^45^. Dual-color dSTORM imaging was employed to reveal the spatially related distributions of EGFR (Fig. 3A) and GlcNAc (Fig. 3B) labeled by Alexa647-linked EGF and Alexa532-linked WGA, respectively, on Vero apical membranes. The colocalized distribution of these two types of clusters on the cell membrane is clearly observed, as shown by the notable yellow regions (Fig. 3C) after the merging treatment. According to the size of the colocalization area, the microdomains were sorted into three types (Fig. 3D) to quantify the colocalization degree by the analysis of their individual percentages (Fig. 3E). For EGFR, the high level of colocalization (69.22 ± 7.28%) and the correlated relationship (16.70 ± 3.20%) suggest that most of the EGFR domains are associated with GlcNAc clusters; similarly, only 16.96 ± 7.26% of GlcNAc clusters are independent of EGFR domains. Additionally, the dual-color dSTORM images of EGFR and GlcNAc were analyzed using the Mander’s coefficient (Fig. S9, Supporting Information). The colocalization of EGFR with GlcNAc is 0.56 ± 0.06, and the colocalization of GlcNAc with EGFR is 0.61 ± 0.09. Altogether, these results obtained from two measurements reveal that most EGFR domains are located inside or in proximity to GlcNAc clusters, suggesting that the carbohydrates-based assemblies are the unique functional domains where membrane functional glycoproteins aggregate to mediate cellular processes.

Next, we preformed the dual-color dSTORM imaging of EGFR and oligosaccharide, by being labeled with Alexa647-linked EGF and Alexa532-linked PHA-L, respectively. The merged images show that plenty of EGFRs are colocalized to oligosaccharides (Fig. S10A, Supporting Information). With Mander’s coefficient test, we find EGFR colocalized with oligosaccharide is 0.50 ± 0.03, and oligosaccharide colocalized with EGFR is 0.55 ± 0.11. These results further demonstrate that most EGFR domains are organized into the common functional platform including different types of carbohydrate clusters.

To further confirm that the carbohydrate cluster is an important platform of multiple functional proteins, we located the EGFR on the cells treated with β-N-acetylglucosaminidase which can digest terminal β-linked N-acetylglucosamine and N-acetylgalactosamine from various glycoconjugates on cell membrane. From Fig. S11, with the disappearance of carbohydrates, a majority of EGFRs cannot locate on the treated membrane by reducing from 348.2 ± 59.1 N/μm^2^ to 77.9 ± 17.7 N/μm^2^, with significant reduction in cluster size (from 0.10 ± 0.01 μm^2^ to 0.04 ± 0.004 μm^2^) and in cluster density (from 0.89 ± 0.14 N/μm^2^ to 0.38 ± 0.10 N/μm^2^), demonstrating that the functional carbohydrate cluster is a fundamental platform where EGFR domains locate. Besides, the similar morphology of EGFRs in dual-color imaging and single-color imaging (Fig. S12, Supporting Information) can further suggest that clustering is a real state for the organization of carbohydrates (given that lectins cross-linked its specific carbohydrate to form artificial clusters during labeling cells, glycoconjugates would alter their native morphologies).

### Colocalization between GlcNAc clusters and band 3 domains

**Figure 4:**
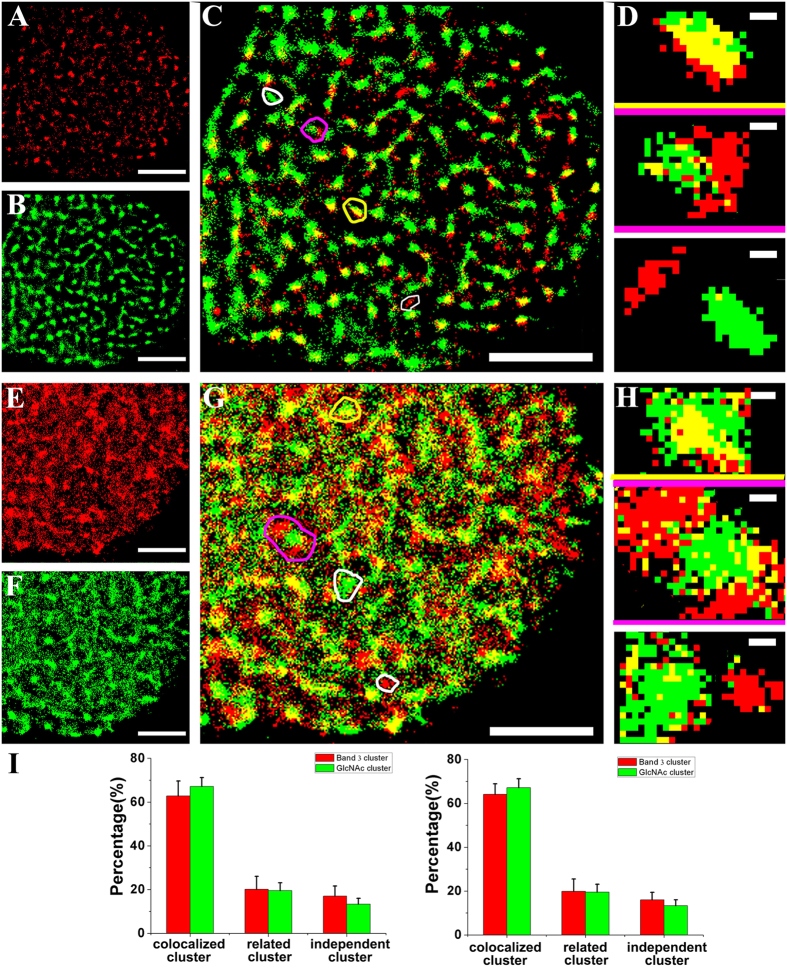
Dual-color dSTORM imaging of band 3 and GlcNAc on Vero apical membranes prepared with complementary protocols. (**A,B**) dSTORM imaging of band 3 and GlcNAc on Vero apical membrane sheets labeled with Alexa647-linked band 3 antibody and Alexa532-linked WGA, respectively. (**C**) The superimposed image of (**A,B**) shows that most of them colocalized with each other. (**D**) The enlarged images illustrate three classification groups of clusters: colocalized clusters (yellow box), correlated clusters (pink box), and independent clusters (white box). (**E–H**) Images corresponding to (**A–D**) of the full cell membrane slightly perforated by saponin. (**I**) Histograms of the numerical percentages of three types of clusters on the membrane sheet (left) and the perforated membrane (right). Scale bars are 5 μm in (**A–C**) and (**E–G**), and 200 nm in (**D,H**). All statistical analyses are acquired from ten cells in three independent experiments.

The erythrocyte anion exchanger (band 3) is commonly expressed in erythroid and the kidney cells and combines with an array of proteins (protein 4.2, ankyrin, and glycolytic enzymes) to form the band 3 complex attached to the cytoskeleton^46^. Thus, bands 3 is selected to investigate the spatial relationship between carbohydrate clusters and functional proteins domains on the cytoplasmic side using dual-color dSTORM imaging. In light of both the large N-terminal domain and short C-terminal tail of band 3 locate in the cytoplasm, two imaging processes were performed for band 3: one in the membrane sheet and another in the full cell with slight perforation mediated by the treatment with saponin, a common chemical permeabilization reagent. dSTORM imaging of band 3 in the membrane sheet (Fig. 4A) shows that most of these proteins exist in clusters. From Fig. 4B, it can be seen that GlcNAc clusters are generally smaller than before, which may result from different labeling conditions. That is, during the preparation process of the membrane sheet, GlcNAc contacted the glass coverslip and therefore was not exposed to the external environment, making some GlcNAc molecules difficult to be labeled. However, this issue does not affect the observation of the positional relationship of GlcNAc with band 3 because the GlcNAc clusters are clearly distinguished. Then, the superimposed images (Fig. 4C), along with three representative groups of clusters (Fig. 4D), display the distribution details of the GlcNAc clusters and band 3 domains. The imaging results on the full cell, either for band 3 (Fig. 4E) or GlcNAc (Fig. 4F), are generally characterized by clustering despite more single localizations. The plausible explanation for such results is that perforating the cell membrane slightly affects cluster morphology. However, it should be mentioned that the colocalization phenomenon is still noticeable in the superimposed image (Fig. 4G). The percentages of the three relationships provide a clue as to the mutual locations between GlcNAc and band 3. It is found that the proportionality of the independent clusters is quite low, regardless of the treatment of the cell membrane. On the membrane sheet, GlcNAc clusters and band 3 domains display percentages of 13.9 ± 4.7% and 17.7 ± 4.6%, respectively. Similarly, on the full cell, only 16.0 ± 3.4% of band 3 domains and 13.4 ± 2.6% of GlcNAc clusters are independent. These results demonstrate that most band 3 domains are located in or associated with GlcNAc clusters. Besides, the colocalization of band 3 and GlcNAc is also demonstrated by the Mander’s coefficient test (Fig. S13, Supporting Information), with 0.51 ± 0.08 for band 3 and 0.54 ± 0.08 for GlcNAc on cell membranes, and 0.67 ± 0.04 for band 3 and 0.71 ± 0.03 for GlcNAc on the full cell with slightly perforation.

In addition, the dual-color dSTORM imaging of band 3 and oligosaccharide was performed on Vero membranes to test their positional relationship. From the merged images and the histogram of Mander’s coefficient (Fig. S14, Supporting Information), it is easy to find that most of them are colocalized with each other, with 0.47 ± 0.04 for band 3 and 0.52 ± 0.08 for oligosaccharide, respectively. Similarly with EGFR, we preformed the dSTORM imaging of band 3 on the membranes treated with β-N-acetylglucosaminidase, and found that localization density on cell membrane decreased from 300.9 ± 18.2 N/μm^2^ to 255.0 ± 16.0 N/μm^2^, and cluster size of band 3 reduced from 0.10 ± 0.01 to 0.05 ± 0.01 μm^2^, indicating that the functional carbohydrate clusters are also related to the proteins domains.

## Discussion

With numerous membrane proteins and lipids (including functional proteins, receptors and glycophosphatidylinositol anchor) being modified carbohydrates, carbohydrates formed on the cell membrane carry the unique information of structure and function. As an indispensable structural component, structural carbohydrates are linked to proteins and lipids to serve for organizational scaffold. With linking to proteins, carbohydrates can mediate the dissolubility and stability of corresponding proteins. Via interacting with carbohydrate-binding proteins, carbohydrates can also direct the glycoconjugate transport, mediate and regulate cellular adhesion and signal transduction. But, their distributed patterns underlying various cellular processes remain unclear. In this work, we systemically investigated the micropatterns of various carbohydrates on Vero apical membranes via dSTORM imaging and confirmed the clustering features of all types of target carbohydrates, with understanding the detailed morphological features of each one, including cluster area size, cluster shape, and cluster density. Based on this result, it can be inferred that, by clustering multiplex glycoconjugates (proteins and lipids) containing the same carbohydrate, appreciable carbohydrate-related aggregates are formed on Vero apical membranes. With their intrinsic clustering patterns, carbohydrates are probably involved in protein sorting on the cell membrane, which is consistent with previous findings^47^,^48^,^49^. Dual color dSTORM imaging between carbohydrates of interest and GlcNAc demonstrates that all target carbohydrates tend to colocalize with GlcNAc clusters, albeit to different extents. Meanwhile, both EGFR domains (membrane receptor) and band 3 domains (channel protein) were revealed to colocalize with GlcNAc clusters and oligosaccharide, as well as the significant changes in distributions of EGFR and band 3 with the digestions of GlcNAcs and GalNAcs, suggesting that carbohydrate clusters indeed exist as functional domains where various correlated glycoproteins are located. As known, the interactions between carbohydrate ligands and corresponding binding proteins are typically low affinity, with Kd values in the μM to mM range. Thus, from our results, we infer that lots of glycoconjugates tend to form clusters where various carbohydrates and proteins are confined, which can intensify the protein-protein interactions to work better for cell processes. Additionally, owing to plenty of functional proteins located into these common platforms, there is no doubt that surface carbohydrate-based connectivity is essential in the control of multiple biological processes^20^,^38^ including cellular signaling^50^ and receptor turnover and endocytosis^51^. Altogether, our findings provide important insights into the systematic organization of different carbohydrates on cell membranes, as well as the spatial relationships of carbohydrate clusters and membrane function proteins. Such information regarding the micropatterning performances of various carbohydrates may shed new light on the organization of the cell membrane and is therefore useful for better understanding the critical role of carbohydrates in various cellular physiological and pathological processes and the development of glycobiology.

## Additional Information

**How to cite this article**: Chen, J. *et al*. Systemic localization of seven major types of carbohydrates on cell membranes by dSTORM imaging. *Sci. Rep.* **6**, 30247; doi: 10.1038/srep30247 (2016).

## Supplementary Material

Supplementary Information
