# Supplementary material for: Systemic localization of seven major types of carbohydrates on cell membranes by dSTORM imaging
Source: Sci Rep. 2016 Jul 25;6:30247. doi: 10.1038/srep30247 (PMC4958959; doi:10.1038/srep30247)
Supplement: Supplementary Information [file srep30247-s1.doc]

**Supporting Information**

**Systemic localization of seven major types of carbohydrates on cell membranes by dSTORM imaging**

*Junling Chen*, *^a^* *^b^ Jing Gao, ^a,b^* *Min Zhang ^a,b^, Mingjun Cai, ^a^ Haijiao Xu, ^a^ Junguang Jiang, ^a^ Zhiyuan Tian, ^c^* and *Hongda Wang * ^a^*

^a^ State Key Laboratory of Electroanalytical Chemistry, Changchun Institute of Applied Chemistry, Chinese Academy of Sciences, Changchun, Jilin 130022, P.R. China.

^b^ University of Chinese Academy of Sciences, Beijing 100049, P.R. China.

^c^ School of Chemistry and Chemical Engineering, University of Chinese Academy of Sciences, Beijing 100049, China

* Email: hdwang@ciac.ac.cn

**1. Determination of the saturated labeling concentrations of various Alexa647-linked lectins.**

To ensure that all examined carbohydrates on the cell membrane were labeled, we applied dSTORM technology to image the distributions of carbohydrates on Vero apical membranes, with increasing labeling concentrations of the corresponding Alexa647-linked lectins. From three representative dSTORM reconstruction images of the nanoscale organization of the example carbohydrate (Sia) at increasing labeling concentrations of Alexa647-linked MAL (Fig. S1A-C), we found that Sia clusters became more apparent as more localizations were imaged. Then, by accounting for the localization density of carbohydrates under increasing concentrations of Alexa647-linked lectin, the saturated value was determined by plotting the concentration gradient curve (Fig. S1D). Similarly, we acquired the labeling curves for all carbohydrates (Fig. S1E), and their corresponding saturated labeling concentrations were ~3 μg/mL of MAL for Sia, ~9.3 μg/mL of PHA-L for oligosaccharides, ~2.88 μg/mL of wheat germ agglutinin (WGA) for GlcNAc, ~12.5 μg/mL of AAA for Fuc, ~7 μg/mL of MNA-M for Man, ~3.75 μg/mL of ECL for Gal, and ~10.5 μg/mL of SBA for GalNAc, respectively.


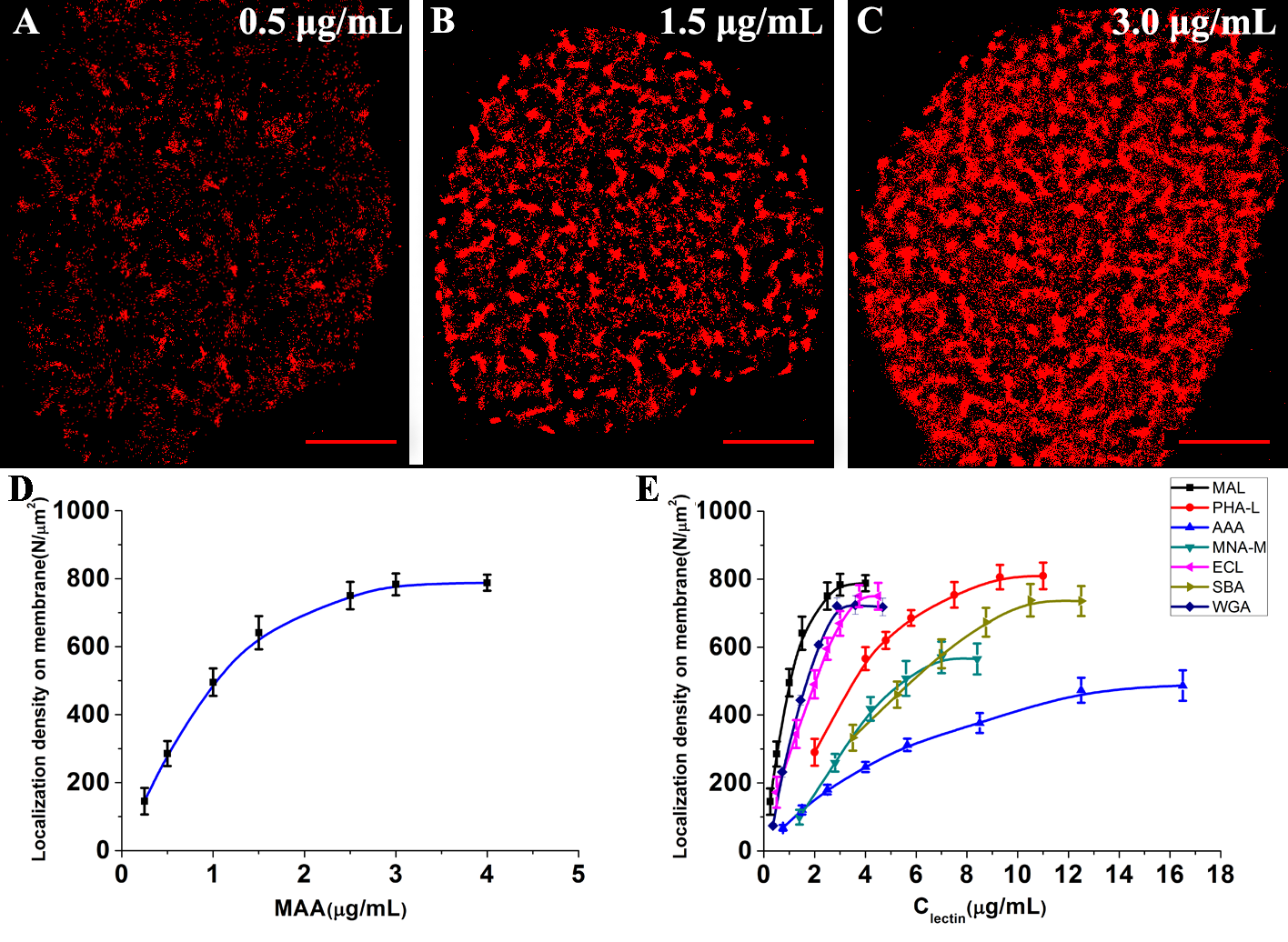


**Figure S1. Determination of the saturated labeling concentrations of Alexa647-linked lectins.** (A-C) Representative dSTORM images of Sia on Vero apical membranes at increasing labeling concentrations: 0.5 μg/mL (A), 1.5 μg/mL (B) and 3 μg/mL (C). (D) The concentration gradient curve, plotted by the change in localization density over the concentration of the Alexa647-linked MAL. (E) The labeling curves for all lectins (MAL, PHA-L, AAA, MNA-M, ECL, SBA, and WGA). Scale bars are 5 μm in A-C.

**2. dSTORM imaging of GlcNAc on live and fixed Vero apical membranes with different fixing time.**

To rule out the cross-linking of lectin to carbohydrates, we performed a series of experiments on imaging the distribution of GalNAc on Vero apical membranes with different fixing time (Figure S2), and found that GlcNAcs mainly formed long dendritic filaments on live cell apical membrane (Fig. S2A). However, most GlcNAc clusters became independent and smaller on the fixed membranes as increasing the fixing time (Fig. S2B-F). Under sufficient fixation, the distribution pattern of GlcNAcs no longer altered again. These results indicate that the carbohydrates on adequately fixed Vero apical membrane can get rid of the cross-linking of lectins.


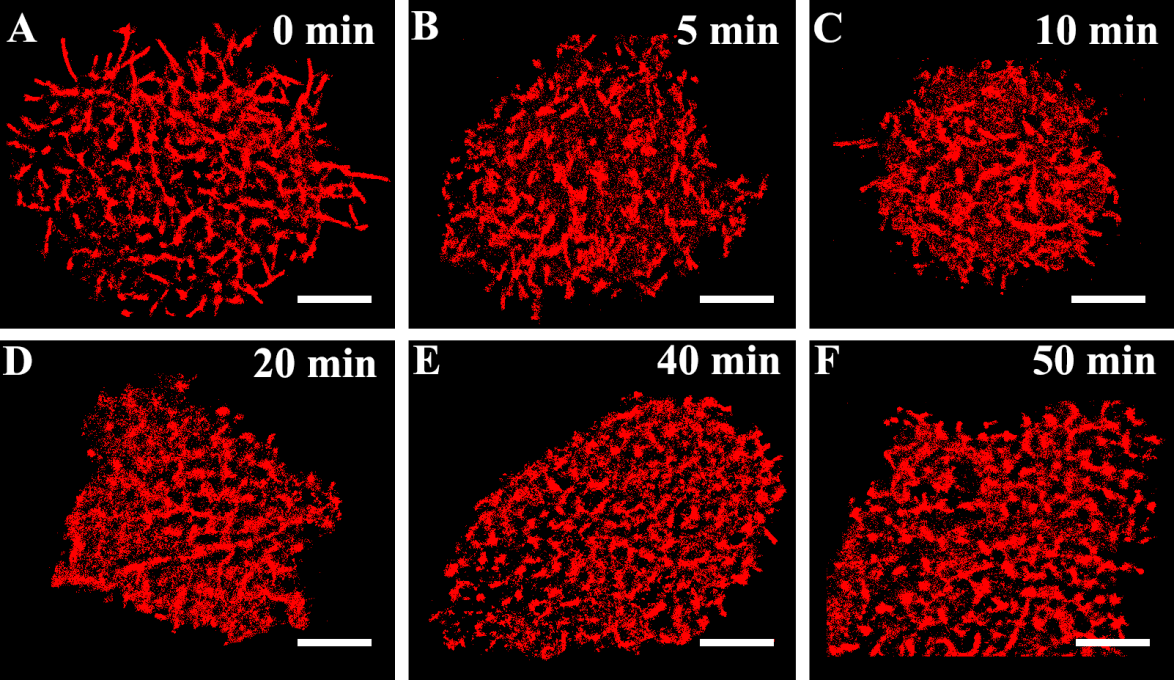


**Figure S2.** The representative dSTORM images of GlcNAc on live (A) and fixed Vero apical membranes with an increasing fixing time. (B) 5 min; (C) 10 min; (D) 20 min; (E) 40 min; (F) 50 min. Scale bars are 5 μm.

**3. dSTORM imaging of seven representative carbohydrates on Vero apical membranes.**

Under the saturated labeling concentration of each Alexa647-lectin, we employed dSTORM to study the spatial organizations of seven types of carbohydrates on Vero apical membranes at the nanometer level. Compared with conventional fluorescence imaging (Fig. S3A-G, upper left), dSTORM imaging (Fig. S3A-G, main parts) exhibited markedly improved resolution. At first glance, clustering was a common and apparent distributed feature for all classes of carbohydrates, with differences in size and shape. The enlarged figures (Fig. S3A-F, lower) further displayed the distinct organizations of different carbohydrates. Generally, Sia and oligosaccharide gathered into large and dense clusters, similarly to GlcNAc; conversely, Fuc existed in the smallest and sparse clusters. Man, Gal and GalNAc, with similar distribution features, were mostly organized into clusters without clear boundaries.

**
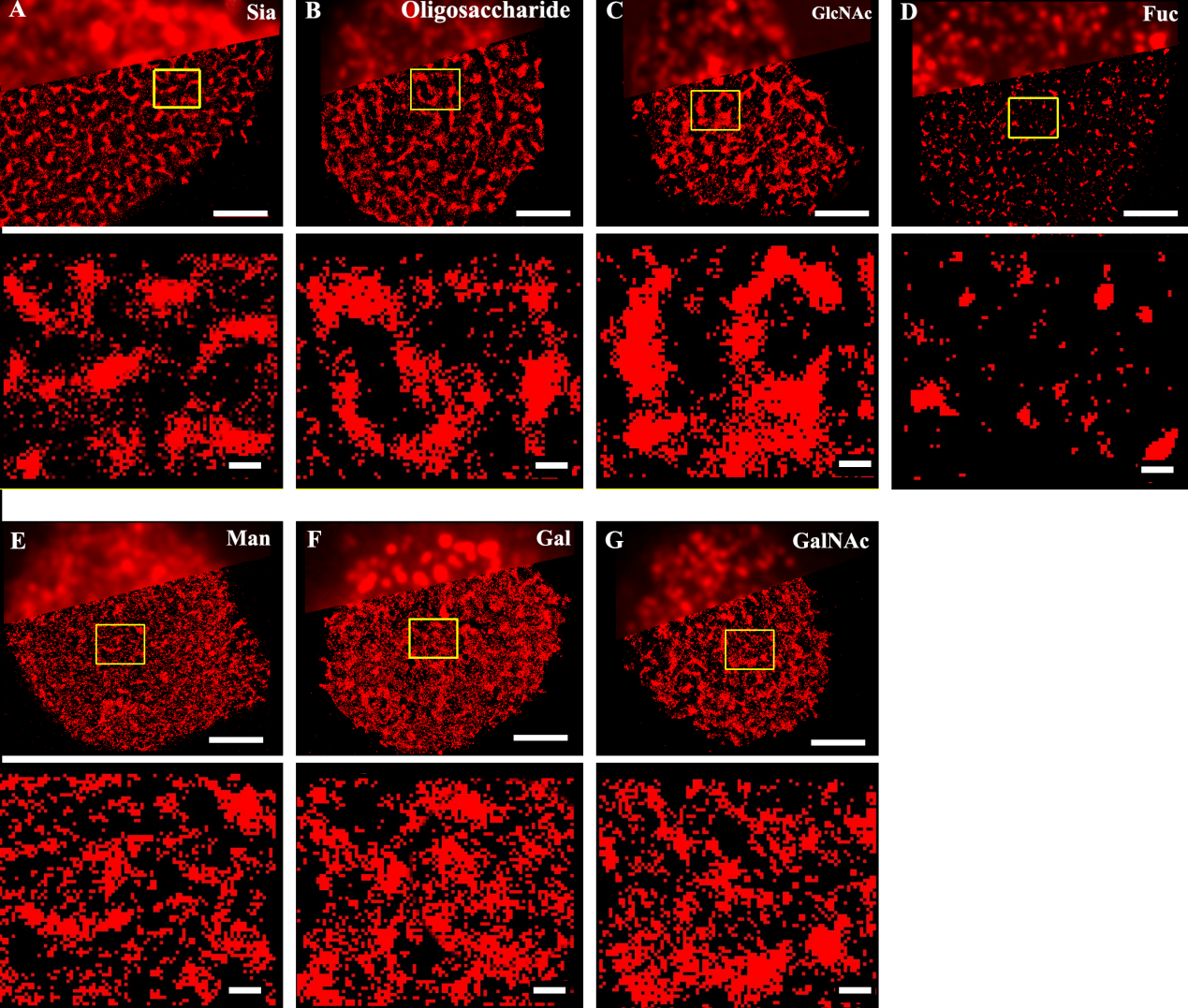
**

**Figure S3. dSTORM imaging of seven types of carbohydrates on Vero membranes by labeling with specific Alexa647-connected lectins**. Sia (A), oligosaccharide (B), GlcNAc (C), Fuc (D), Man (E), Gal (F), and GalNAc (G). Compared with conventional fluorescence images of each type of carbohydrate (A-F, upper-left), the corresponding dSTORM images (A-G, upper) depict much sharper distribution patterns of carbohydrates at an improved resolution. (A-G, lower) The enlarged images of the boxed regions clearly illustrate the detailed features of the clusters. Scale bars are 5 μm in the upper images of A-F and 500 nm in the lower images of A-F.

**4. Image-based cluster analysis and BDSCAN cluster analysis of Fuc clusters.**


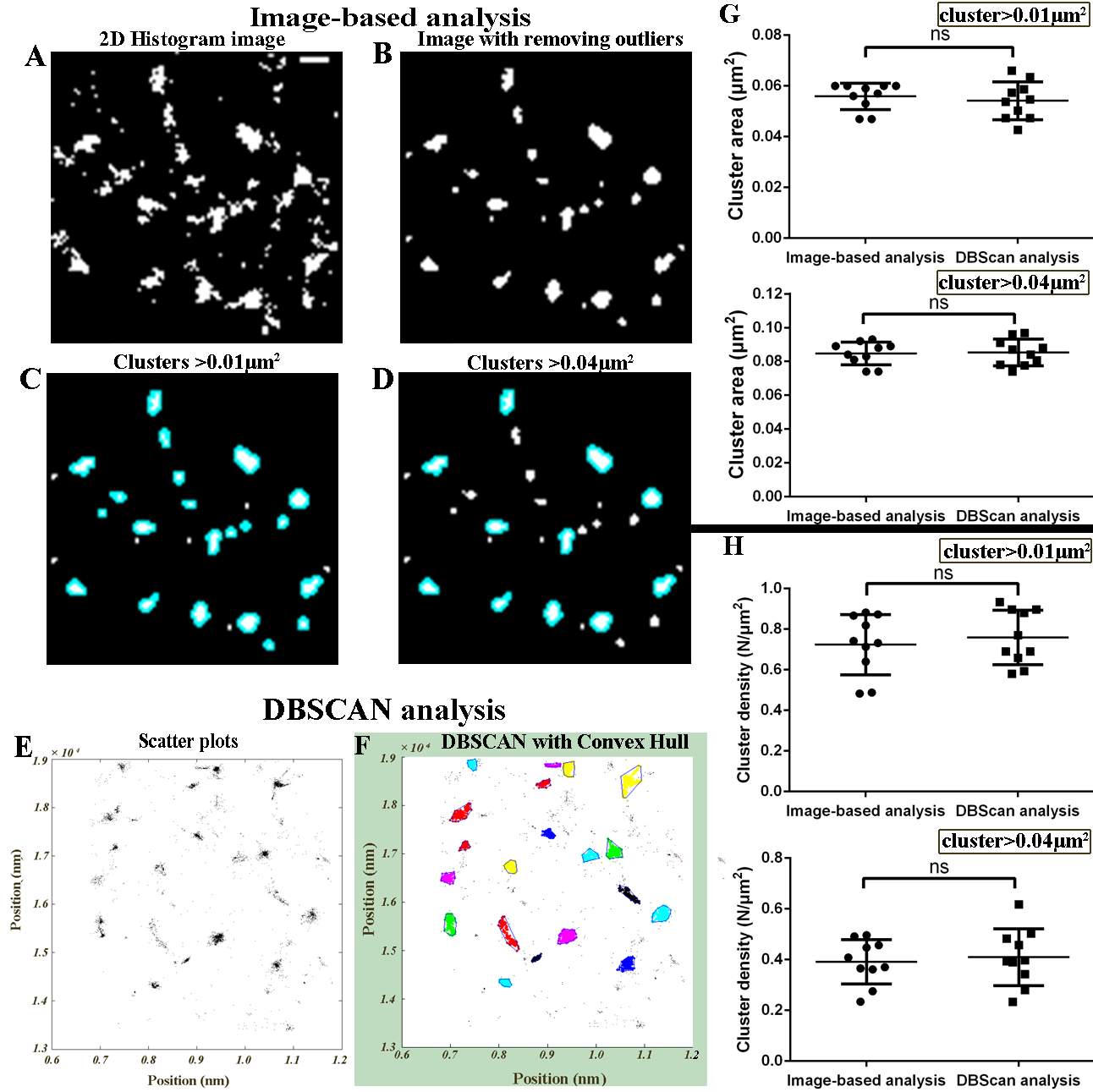


**Figure S4. Fuc clusters were characterized by image-based analysis and DBSCAN analysis. (**A) The original reconstructed dSTORM image in view mode of 2D histogram. Scale bar is 2 μm. (B) The binary image treated with "*Remove Outliers*". (C and D) The images of quantified clusters (outlined in cyan). (E) The dSTORM image generated as scatter plots in MatLab. (F) The qualified clusters were identified by DBSCAN analysis. (G and H) The compared analyses of average cluster area (G) and cluster density (H) from these two analysis methods, with setting threshold of cluster size is 0.01 μm^2^ (upper image) and 0.04 μm^2^ (lower image). All statistical analyses were acquired from ten cells from three independent experiments. Data are the mean (long line) ± standard deviation (s.d.) (short line). 'ns' means no significance, analysis of variance by the two-tailed unpaired t-test.

**5. BDSCAN cluster analysis of GlcNAc cluster with setting different search radius (ε) for cluster identification.**

**
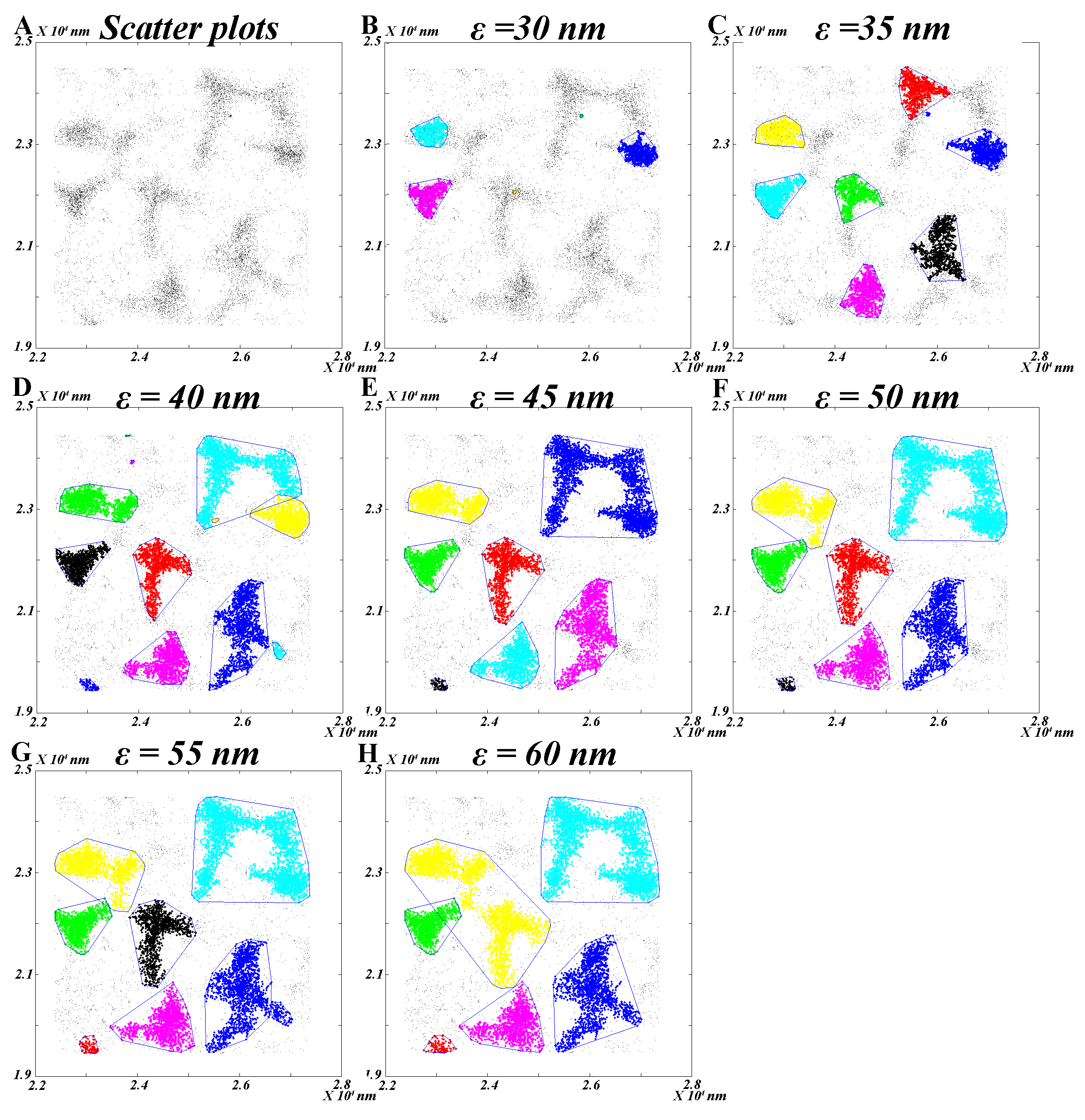
**

**Figure S5.** (A) dSTORM image of the distribution of GlcNAcs on Vero apical membrane in view mode of scatter plots. (B-H) The images of quantified clusters with convex hull identified by DBSCAN analysis with setting different search radius (*ε*), including *ε*=30 nm (B), *ε*=35 nm (C), *ε*=40 nm (D), *ε*=45 nm (E), *ε*=50 nm (F), *ε*=55 nm (G), and *ε*=60 nm (H).

**6. Image-based cluster analysis of the distribution patterns of all types of carbohydrates on Vero apical membranes.**


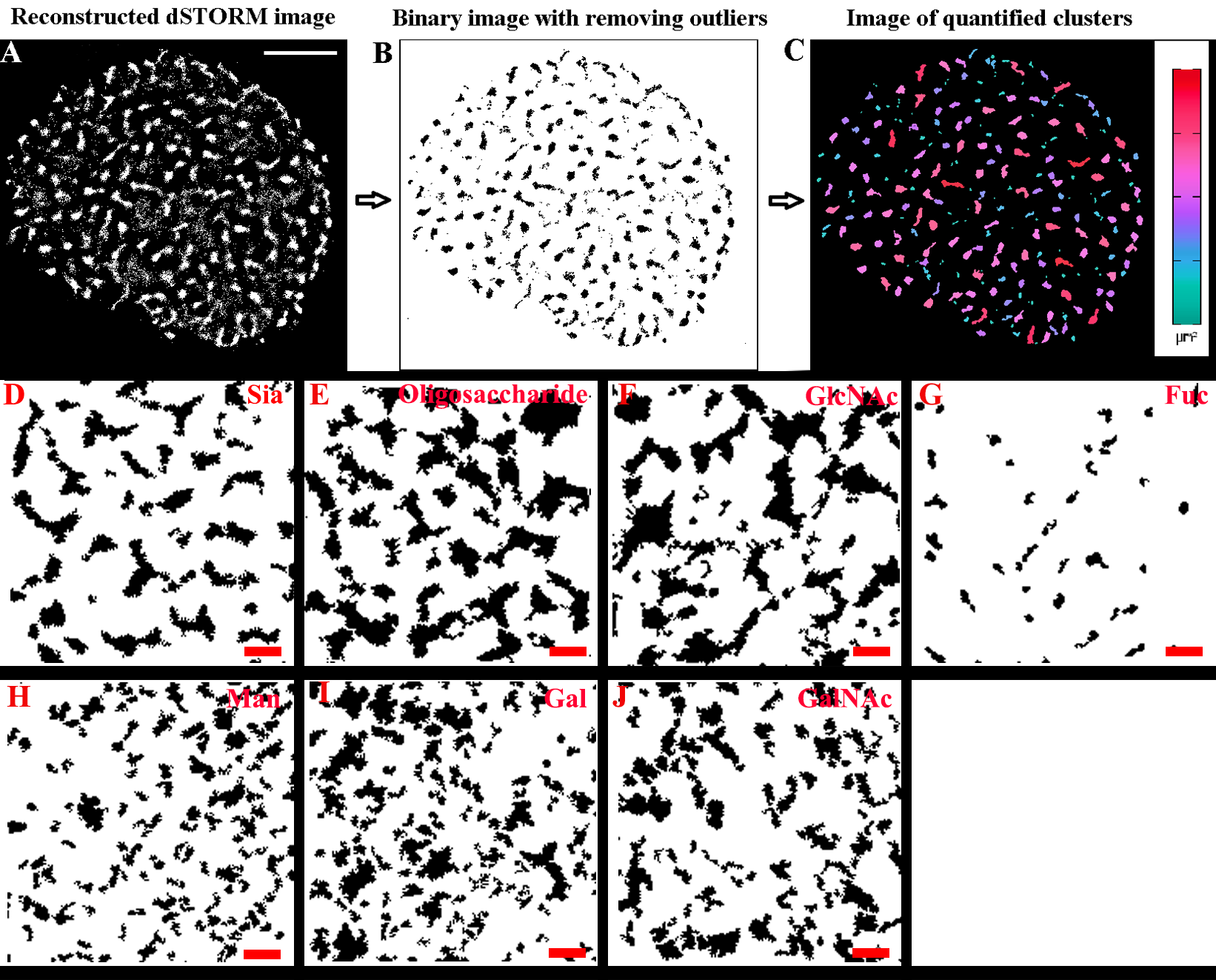


**Figure S6. Image-based cluster analysis of seven types of carbohydrates.** (A) Representative reconstruction dSTORM image of the organization of Sia on Vero membrane. (B) The binary image where single localizations removed by applying "*Remove Outliers*". (C) Distribution of the qualified clusters abstracted from Figure B. (D-J) Representative images of the distribution of the qualified clusters of different carbohydrates. Sia (D), oligosaccharide (E), GlcNAc (F), Fuc (G), Man (H), Gal (I), GalNAc (J). Scale bars are 5 μm in A-C and 1 μm in D-J.

**7. Mander's coefficient test of colocalization of multiple carbohydrates and GlcNAc.**


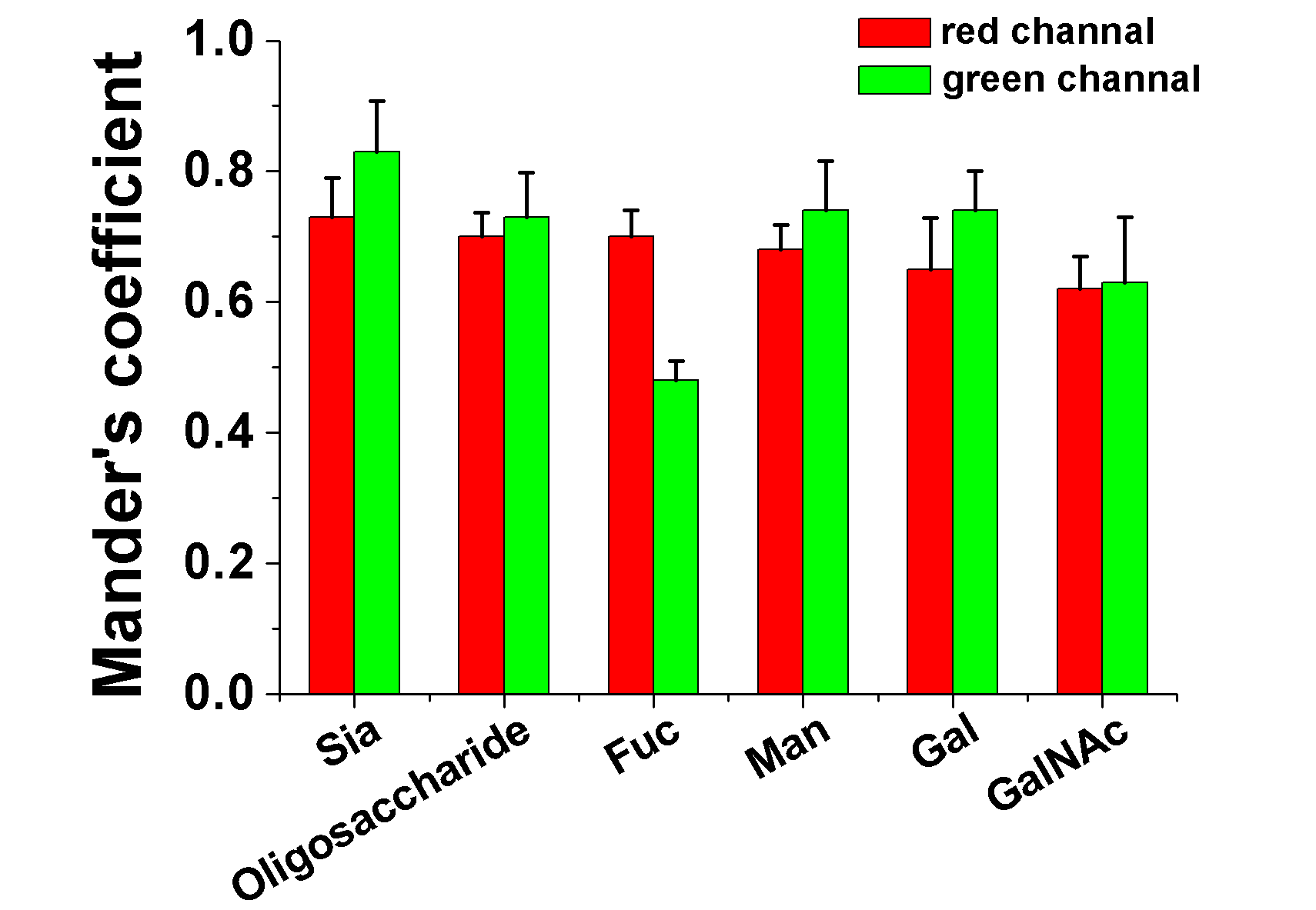


**Figure S7.** The histogram of Mander's coefficients of red channel and green channel to test the colocalization of examined carbohydrates with GlcNAc. All statistical analyses are acquired from more than ten cells (mostly 10-15 cells) in three independent experiments. Data are the mean ± standard deviation (s.d.).

**8. Colocalization analysis of the simulated data.**


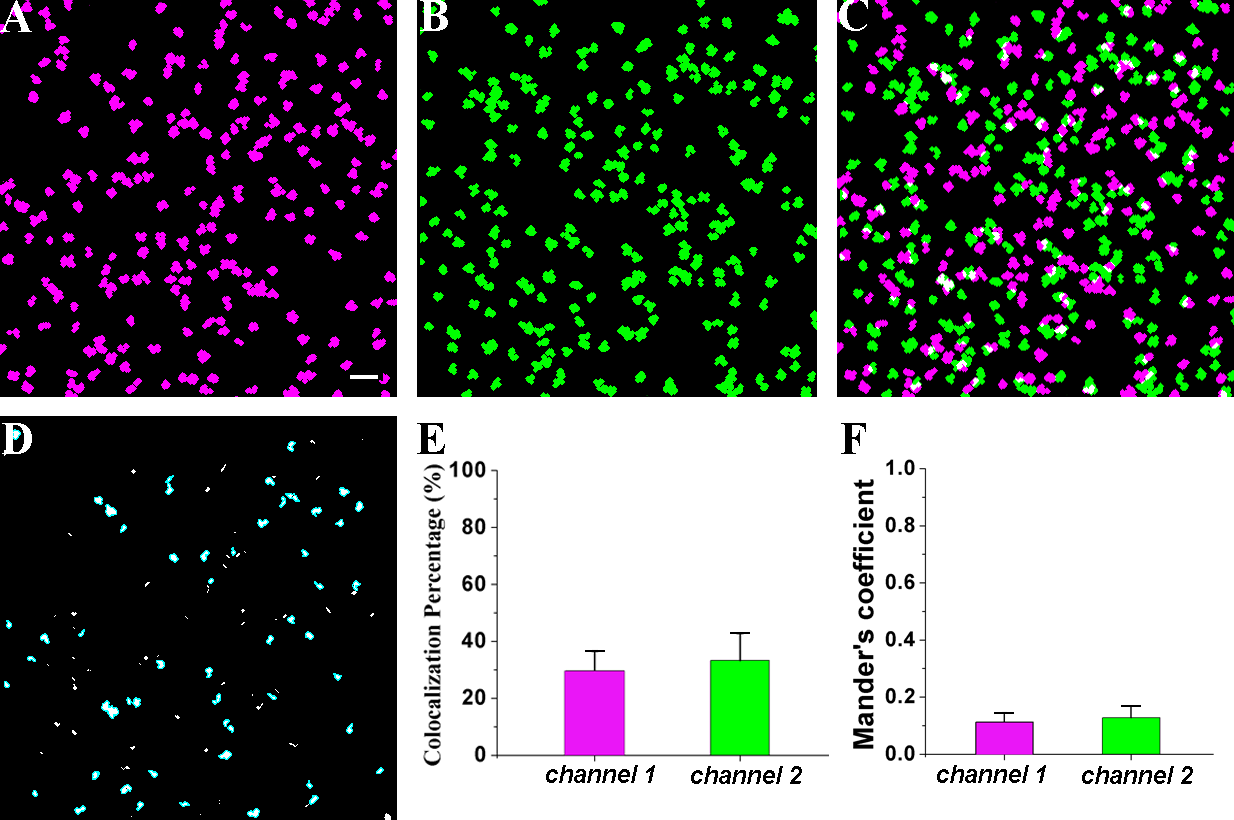


**Figure S8. The simulated data of random distribution of domains was analyzed by two colocalization analysis methods.** (A and B) The representative two channel images where simulated domains randomly distribute. Scale bar is 2 μm. (C) The merged image of Figure A and B. (D) The image of the colocalization region, with the quantified clusters being outlined in cyan (>0.02 μm^2^). (E) The percentages of colocalized clusters to total clusters in two channels. (F) The Mander's coefficients characterizing the colocalization of two channels. All statistical analyses were acquired from ten simulated images. Data are the mean ± standard deviation (s.d.).

**9. Mander's coefficient test of colocalization of EGFR between GlcNAc.**

**
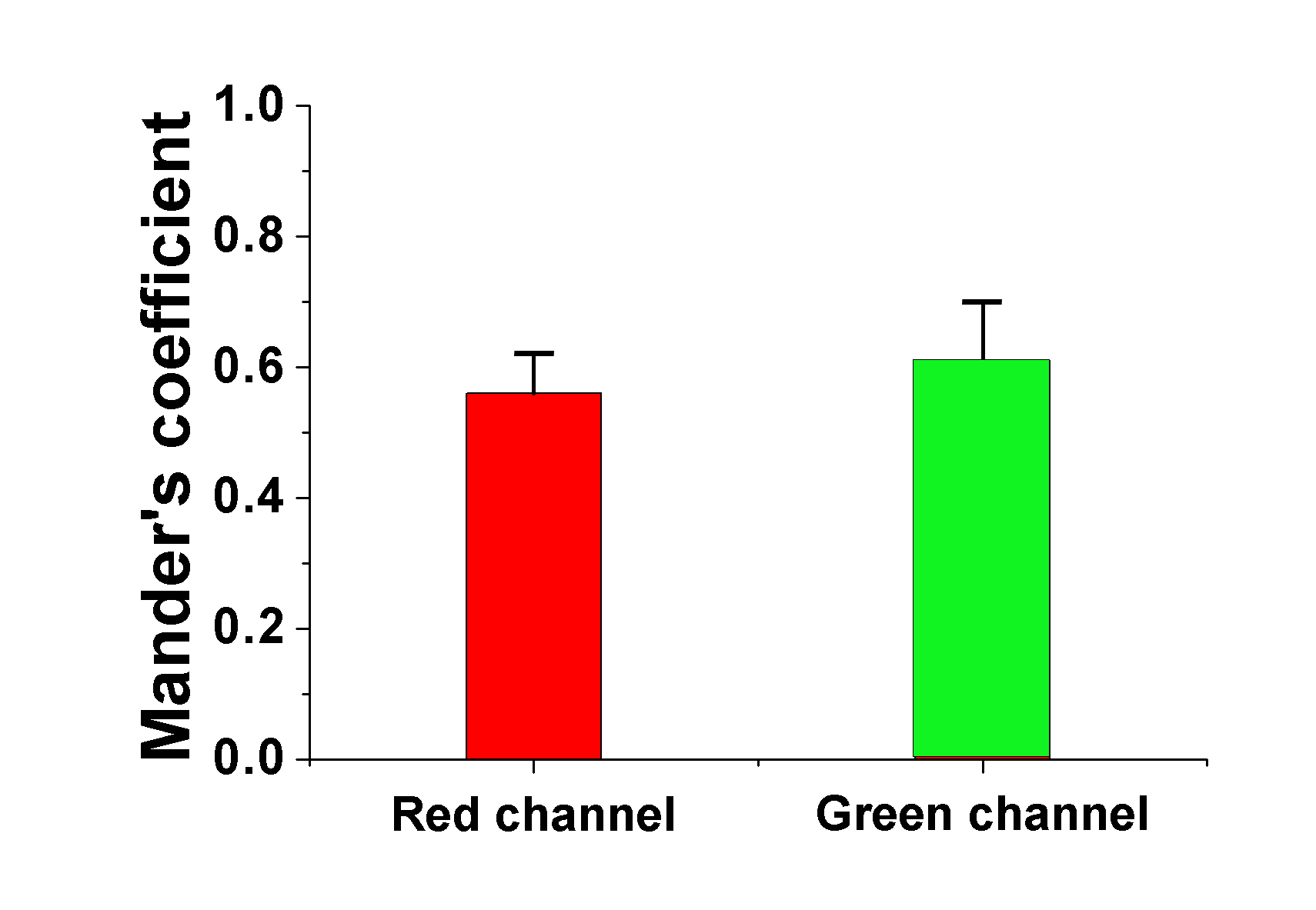
**

**Figure S9.** The statistical analyses of Mander's coefficients in two channels to test the colocalization of EGFR to GlcNAc. All statistical analyses are acquired from ten cells in three independent experiments. Data are the mean ± standard deviation (s.d.).

**10. Dual-color imaging and colocalization analysis of EGFR and oligosaccharide.**

**
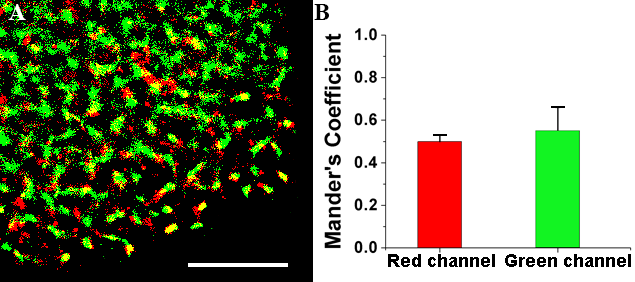
**

**Figure S10.** (A) The merged images of the distributions of EGFR (red color) and oligosaccharide (green color), with labeling of Alexa647-linked EGF and Alexa532-linked PHA-L, respectively. (B) The histogram of Mander's coefficient in two channels. All statistical analyses are acquired from ten cells in three independent experiments. Data are the mean ± standard deviation (s.d.).

**11. The changed morphology of EGFR in the membranes treated with β-N-acetylglucosaminidase compared with that on normal cell membranes.**

**
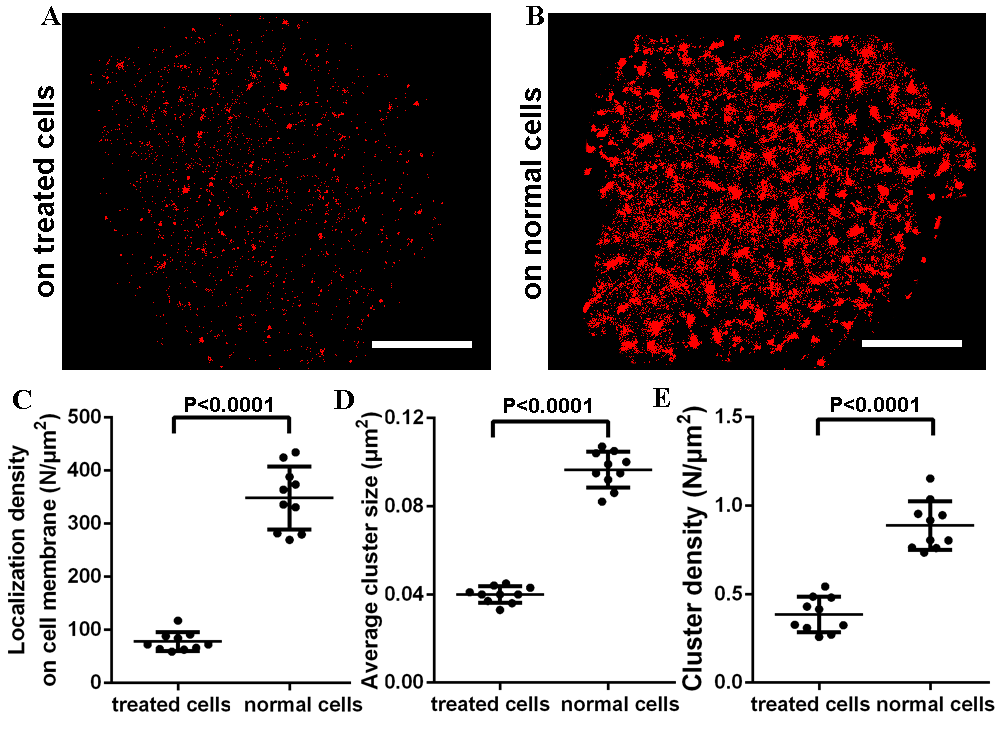
**

**Figure S11. d**STORM imaging of the distributions of EGFRs on the treated membranes with β-N-acetylglucosaminidase (A) and the normal cell membranes (B), scale bars are 5 μm. (C-E) Statistical analyses of the changed morphologies of EGFR on the treated membranes, including the localization density on cell membrane (C), the average cluster size (D) and the cluster density (E). All statistical analyses were acquired from ten cells in three independent experiments. Data are the mean (long line) ± standard deviation (s.d.) (short line). Analysis of variance by the two-tailed unpaired t-test.

**12. The compared dSTORM images of EGFR in Vero apical membranes between dual color imaging of EGFR and GlcNAc and single color imaging of EGFR.**

Given that lectin cross-linked its specific carbohydrate to form various clusters during staining cells, the morphology of glycoconjugates would be altered by reorganizing on the cell membrane. That is, if carbohydrate clusters were indeed formed by the cross-linking of lectin, the distribution of EGFR in Vero apical membrane labeled alone with Alexa647-linked EGF would differ from that labeled with Alexa647-linked EGF followed by staining cells with Alexa532-linked WGA. Here, we compared the organizations of EGFR in Vero apical membrane between single color imaging of EGFR (Fig. S12A) and dual color imaging of EGFR and GlcNAc (Fig. S12B), and found that the morphologies of EGFR in Vero apical membranes under these two labeling conditions were very similar. This result further suggests that carbohydrates were undoubtedly distributed in clustering state on Vero apical membrane.


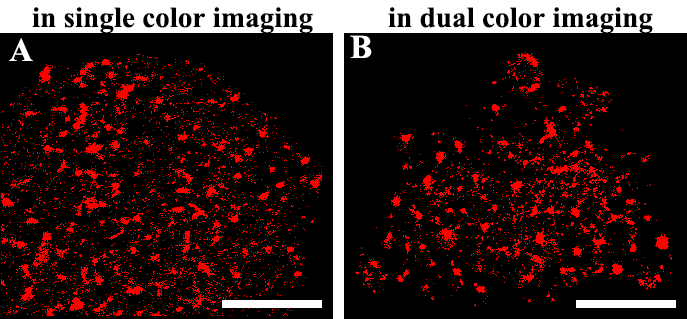


**Figure S12.** The dSTORM imaging of EGFR in Vero apical membranes in single color imaging (A) and dual color imaging of EGFR and GlcNAc (B) where EGFR is labeled with Alexa647-linked EGF before staining GlcNAc with Alexa532-linked WGA. Scale bars are 5 μm.

**13. Mander's coefficient colocalization analysis of band 3 and GlcNAc.**

**
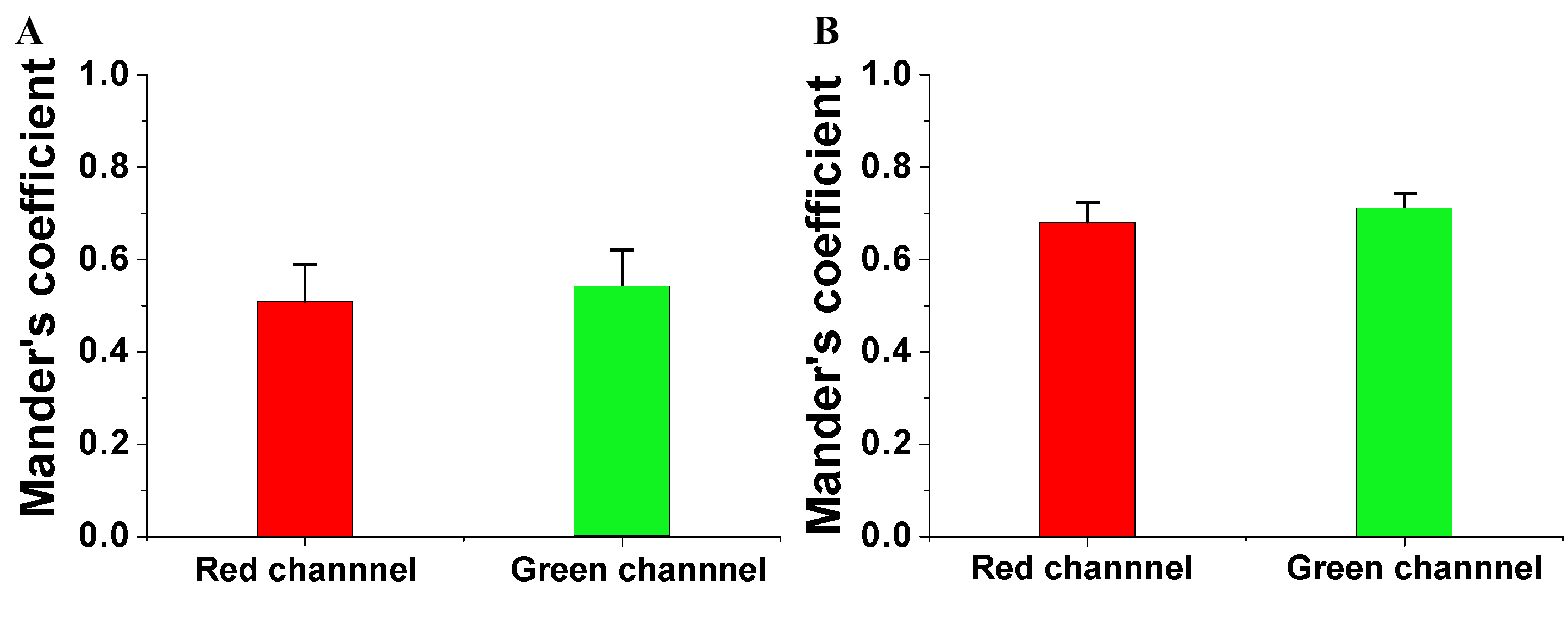
**

**Figure S13.** The statistical analyses of Mander's coefficients in two channels to characterize the colocalization of band 3 and GlcNAc on Vero apical membrane sheets (A) and on cell membrane slightly perforated by saponin (B). All statistical analyses were acquired from ten cells in three independent experiments. Data are the mean ± standard deviation (s.d.).

**14. Dual-color imaging and colocalization analysis of band 3 and oligosaccharide.**

**
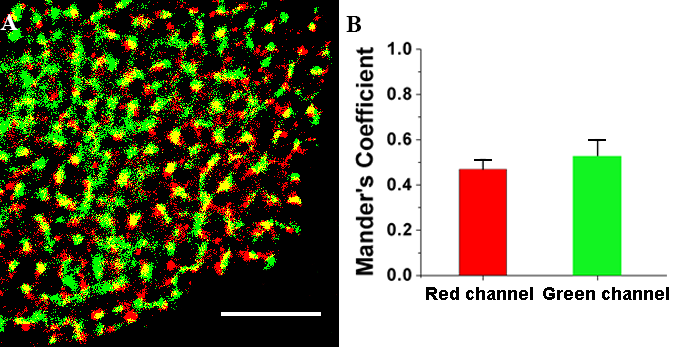
**

**Figure S14.** (A) The merged images of the distributions of band 3 (red color) and oligosaccharide (green color) on Vero apical membrane sheets, with labeling of Alexa647-linked BIII 136 and Alexa532-linked PHA-L, respectively. (B) The histogram of Mander's coefficient in two channels. All statistical analyses were acquired from ten cells in three independent experiments. Data are the mean ± standard deviation (s.d.).

**15. The changed morphology of band 3 in cell membranes treated with β-N-acetylglucosaminidase compared with that in normal cell membranes.**

**
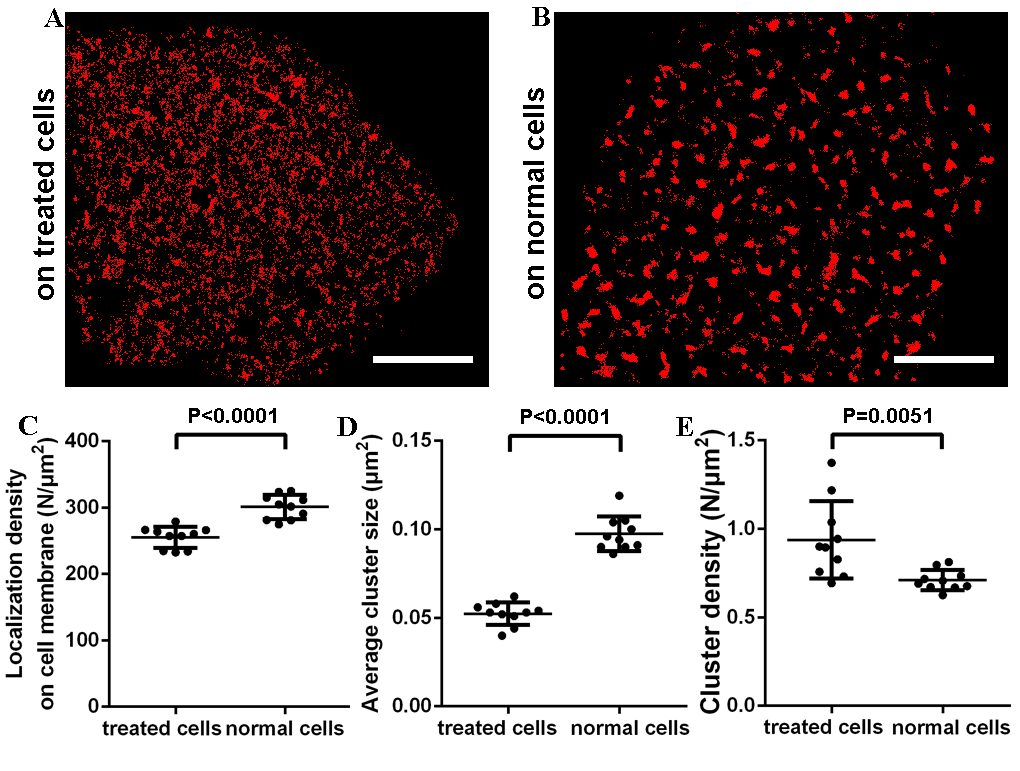
**

**Figure S15.** dSTORM imaging the distributions of band 3 on the membranes treated with β-N-acetylglucosaminidase (A) and the normal cell membranes (B), scale bars are 5 μm. (C-E) Statistical analyses of the changed morphologies of band 3 on the treated membranes, including the localization density on cell membrane (C), the average cluster size (D) and the cluster density (E). All statistical analyses were acquired from ten cells in three independent experiments. Data are the mean (long line) ± standard deviation (s.d.) (short line). Analysis of variance by the two-tailed unpaired t-test.

**16. Material and Methods**

**16.1 Cell culture**

Vero cells, African green monkey kidney cells, from the Shanghai Institute of Biological Sciences, were cultured in a 5% CO_2_ environment at 37°C in minimum essential medium (MEM, HyClone) supplemented with 10% fetal bovine serum (FBS, HyClone), 100 U/mL penicillin and 100 μg/mL streptomycin. For dSTORM imaging, the cells were cultured on clean cover slips (22 mm × 22 mm, Fisher) that is placed in culture dish with the medium for ~24 hours to achieve ~60-70% confluence.

**16.2 Sample preparation**

**16.2.1 Preparation of specific probes for labeling different carbohydrates and membrane proteins**

Although several hundred distinct monosaccharides are known to occur in nature, only a small number of these are commonly found in animal glycans. Hence, in our research, several types of common and representative monosaccharides and a oligosaccharide chain were selected: Hexoses (Galactose (Gal), and Mannose (Man)), Hexosamines (N-acetylgalactosamine (GalNAc)), Deoxyhexoses (L-fucose (Fuc)), [Sialic acids](http://www.ncbi.nlm.nih.gov/books/n/glyco2/glossary/def-item/glossary.gl1-d164/) (Sia), and Complex-type N-glycans (pentasaccharide sequence Galβ1-4GlcNAcβ1-2(Galβ1-4GlcNAcβ1-6) Manα1-R (the so-called '2,6-branch')). Then, according to the binding specificity between the carbohydrates and lectins (refer to the manufacturer’s introduction), six specific lectins, MAL for Sia (from Sigma), and PHA-L (from EY laboratories) for 2, 6-branch, AAA (from Sigma) for Fuc, ECL (from Sigma) for Gal, SBA (from Sigma) for GalNAc, and MNA-M (from EY laboratories) for Man, were linked to Alexa647 or Alexa532 to act as unique probes for imaging the corresponding carbohydrates.

The detailed procedure of preparing specific fluorescent probes for each type of carbohydrate is the same as in previous research on GlcNAc [^1^](#_ENREF_1). With similar protocols, EGF (from PeproTech) or band 3 monoclonal antibody (BIII 136, from Santa Cruz) was conjugated to Alexa647 to specifically label membrane proteins (EGFR or band 3).

**16.2.2 Single color dSTORM imaging of carbohydrates**

The cultured cells were first to wash 3 times with 1× PBS, then they are fixed in 1 mL of 4% paraformaldehyde (PFA) in 1× pre-warmed PBS at 37°C for 40 min (similar to the successful protocol of sample preparation in the previous imaging of GlcNAc). The cells were stained with Alexa647-conjugated lectin solution (50 μL) at 4°C for 10 min after washing 3 times with 1× PBS. Then, the cells were washed 4 times with 1× PBS to remove the excess solution.

Before imaging, with dropping ~30 μL of imaging buffer (containing 140 mM beta-mercaptoethanol (βME), 0.5 mg/mL of glucose oxidase and 40 μg/mL of catalase) on a microscope slide (24 mm × 50 mm, Fisher), the small coverslip that the seeded cells were cultured on was gently sealed onto the microscope slide using nail polish.

For dual-color dSTORM imaging of the carbohydrate of interest and GlcNAc, the cultured cells were fixed with the above protocols, then cells were labeled simultaneously with the specific lectins of the carbohydrates of interest with respective saturating concentrations.

**16.2.3 Dual-color dSTORM imaging of EGFR and GlcNAc**

For dual-color dSTORM imaging carbohydrates and EGFR, cultured cells were first washed and fixed with the same above protocol; then, the cells were blocked by 3% BSA for 20 min, labeled by Alexa647-linked EGF at 4°C for 20 min. After washing 4X with PBS, the cells were stained with Alexa647-conjugated WGA at 4°C for 10 min, and this was followed by removing the excess lectin with 4 washes with PBS; before imaging, the slides were sealed as described above.

**16.2.4 Dual-color dSTORM imaging of band 3 and GlcNAc**

Because band 3 and GlcNAc are located on the intra-membrane and extra-membrane, respectively, we performed two complementary labeling treatments. One was imaging on the apical membrane sheet gained by carefully tearing the full cells, thus exposing the binding sites of band 3. The other was imaging on the perforated full cell by treating with 0.005% saponin (from Sigma) for 30 min, which is a common protocol for staining cytoplasmic proteins. Apart from this difference, the remaining procedures were the same, including fixation with paraformaldehyde, blocking with 3% BSA, washing with PBS, staining band 3 using the band 3-specific antibody conjugated with Alexa647, labeling GlcNAc with Alexa532-linked WGA, and sealing.

**16.3. dSTORM imaging**

dSTORM imaging was performed on an inverted Nikon Ti-E microscope with a 100 × 1.49 NA TIRF lens (Nikon, Japan), as well as an objective-type TIRF illumination. The example was imaged in a buffer containing 140 mM beta-mercaptoethanol (βME), 0.5 mg/mL of glucose oxidase and 40 μg/mL of catalase with excitation of a 640 nm laser (single-color imaging) and 532 nm laser (dual-color imaging). With using an excitation filter (ZET532/647x, 25 mmR, Chroma), a dichroic mirror (ZT532/647rpc, 25.5 mm × 36 mm × 1 mm, Chroma) and an emission filter set (ZET532/640m, 25 mmR, Chroma), the dual-color imaging was first performed with excitation of 640 nm laser to acquire a imaging sequence, then, with adding a band pass emission filter (FF01-559/34-25, semrock) to avoid the color-crosstalk of Alexa647, the example was imaged with excitation of 532 nm laser. A time series of 5000 frames (according the trend that the GlcNAc cluster changes with the increasing frame number in the study of GlcNAc ) per cell was recorded at a rate of 25 Hz by an electron multiplying charge coupled device (EMCCD, Photometrics, Cascade II) camera, with the pixel size of 160 nm. During the acquisition time, the sample was stabilized by two clips to reduce the possibility of x-y drift, meanwhile, we used tetra-speck microspheres (100 nm diameter, Invitrogen) as fiducial markers to correct the x-y drift of the sample and the optical registration between Alexa647 and Alexa532 channels for dual color; the z-drift was eliminated by a focus lock.

**16.4. Data analysis**

**16.4.1 Reconstruction of dSTORM image'**

To analyze the dSTORM data, firstly, a sequence of frames (a TIFF stacks) was analyzed by QuickPALM in Image J (developed by National Institutes of Health (NIH)) to reconstruct a dSTORM image. After a pre-process of background subtraction, fluorescence peaks were identified in each frame and least-squares fitting was performed with an elliptical Gaussian function to localize the positions of particles, with setting the minimum SNR (commonly 6 or 8) and maximum FWHM (commonly 3). Then, the centroid positions of peaks were determined and a localization data of single fluorescent molecules was obtained with rejecting the poor fit and asymmetric PSFs. Finally, a reconstructed image was generated as 2D histogram (this mode for view the morphologies of carbohydrates or proteins) or 2D Gaussian rendering of molecular positions (2D particle intensity-8 bit, this mode for Mander's coefficients analysis of colocalization) or scatter plots (this mode for analysis the localization density).

**16.4.2 Determining the saturated labeling concentrations of different carbohydrates.**

With acquiring the scatter plots of the localizations in MatLab (The Mathworks, Natick, MA) by importing the coordinate list obtained by QuickPALM, we can acquire the total number of localizations in the total cell membrane or the region of interest (ROI) after removing the localizations distributed outside the cell membrane or the ROI. Via measuring the definitive size of cell membrane or the ROI with application of the "*Analyze*" in ImageJ, we finally calculated the localization density on cell membrane or the ROI. Thus, the localization densities under different labeling concentrations were generated to plot a labeling curve showing the saturated value.

**16.4.3 Calculation of the ratio of the number of localizations in clusters to the total localizations on the cell membrane**

Similarly with the method of calculation of the localization density on cell membranes, the scatter plots was generated by importing total coordinates of localizations into MatLab. We selected boxed region (2×2-3×3 μm) and obtained the total number of localizations in this region, then, with application of the "*Single L*" (a custom written code in MatLab), we extracted clusters without single localizations and acquired the total number of localizations in cluster. Thus, the ratio of the number of localizations in clusters to the total localizations on the cell membrane can be further calculated.

**16.4.4 Cluster analysis**

DBSCAN (Density Based Spatial Clustering of Applications with Noise) was a common algorithm used to describe clustering in the dSTORM dataset and implemented on the dSTORM localizations as previously described[^4^](#_ENREF_4), with defining search radius for cluster identification (*ε*) and minimum localizations/cluster. During DBSCAN analysis of Fuc in the *SuperCluster* (Jan. 2014 release, a custom program written in MatLab)[^5^](#_ENREF_5), *ε* was set to 45-50 nm and the minimum was set to 20 localizations per cluster. In analysis of GlcNAc, *ε* was set to 30-60 nm and the minimum was set to 20 localizations per cluster. Owing to the large cluster size and irregular shape of GlcNAc clusters, DBSCAN is difficult to accurately identify the GlcNAc clusters (Figure S5). Thus, DBSCAN analysis is not suitable for the other types of carbohydrates which mostly distributed in large clusters with irregular shape.

About image-based cluster analysis of carbohydrate cluster, the method was similar as that for GlcNAc[^1^](#_ENREF_1), seen from Figure S6, With applying "*Remove Outliers"*, we can obtain a clear image where clusters markedly distribute. Then, with converting it to binary image, we implemented *"Analyze Particles"* in ImageJ to analyze the qualified clusters (sometimes independent clusters need to be delimitated by cutting off slight connectivity (*i.e.,* 4- and 8-connected)) and capture some parameters for characterizing each cluster, such as cluster area size, perimeter, and circularity, as well as summary data, including the total number of clusters and the total area of clusters on the examined cell membrane.

**16.4.5 Colocalization analysis**

About the percentage of colocalized clusters which characterizes the colocalization of two types of particles, with a similar method of the cluster analysis in ImageJ, we were first to calculate the total number of clusters in each channel in the same ROI, respectively. Then, we abstracted the colocalized regions from the merged image after adjusting the "*Color Threshold*", and acquired the total number of clusters (>0.02 μm^2^) which represent the colocalized clusters and clusters (<0.02 μm^2^) represent the co-related clusters via "*Analyze Particles*". Thus, we can further calculate percentages of these three classes of positional relationships (including colocalization, co-relation and independence).

About the Mander's coefficient characterizing the colocalization, we imported the reconstructed dSTORM images in two channels (view mode in 2D particle intensity-8 bit) to the Image-Pro Plus (IPP, developed by Media Cybernetics) and measured the Mander's coefficient of two channels via "*co-localization*" in the ROI or entire cell.

We generated the simulated data by using the domain simulation in the *SuperCluster*. The randomized images representing two channels were created by using the following parameters: 10 particles per domains; 0.5 domains/μm^2^；2D Gaussian sigma for domain size was 100 nm, 10 localizations/molecule, 20 nm localization error; 25×25 μm image. Then, we used the two above methods to calculate the percentage of colocalized clusters and Mander's coefficient as a control to compare the colocalized features of our experimental data.

**References**

1 Chen, J. *et al.* Revealing the carbohydrate pattern on a cell surface by super-resolution imaging. *Nanoscale* **7**, 3373-3380, (2015).

2 Evans, L., Hammer, J. & Bridgman, P. Subcellular localization of myosin V in nerve growth cones and outgrowth from dilute-lethal neurons. *Journal of Cell Science* **110**, 439-449 (1997).

3 Koester, S. K. & Bolton, W. E. Intracellular markers. *Journal of immunological methods* **243**, 99-106 (2000).

4 Ester, M., Kriegel, H.-P., Sander, J. & Xu, X. *Kdd.* **96**, 226-231 (1996).

5 Itano, M. S. *et al.* Super-resolution imaging of C-type lectin spatial rearrangement within the dendritic cell plasma membrane at fungal microbe contact sites. *Frontiers in physics* **2** (2014).
